# Supplementary figures and images for: Accounting for Experimental Noise Reveals That mRNA Levels, Amplified by Post-Transcriptional Processes, Largely Determine Steady-State Protein Levels in Yeast
Source: PLoS Genet. 2015 May 7;11(5):e1005206. doi: 10.1371/journal.pgen.1005206 (PMC4423881; doi:10.1371/journal.pgen.1005206)

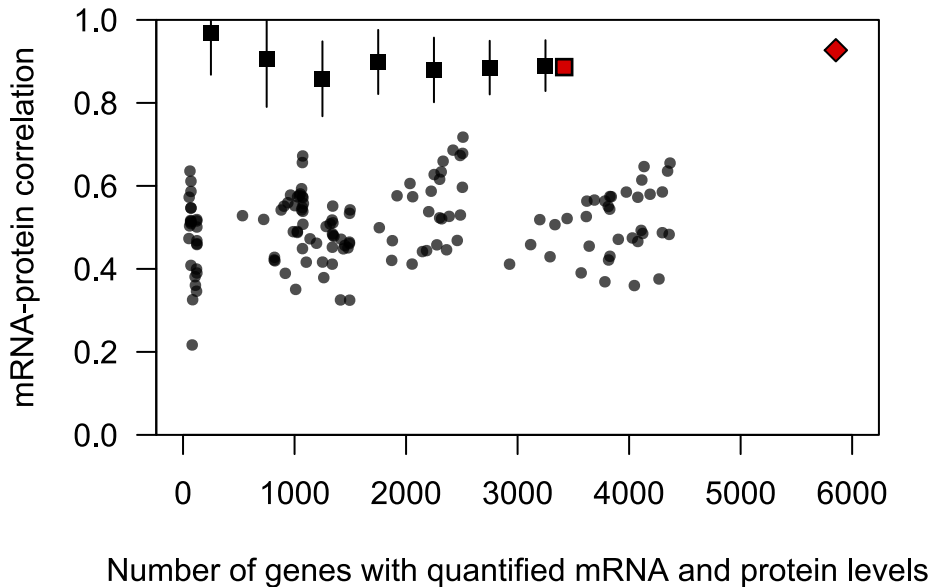

Supplement: S1 Fig — The results of Spearman’s correction on the largest set of paired datasets (red square) and structured covariance model (SCM) fitting (red diamond) are provided for reference. Labels as in Fig 2A. (PDF) [file pgen.1005206.s001.pdf]

Proportion

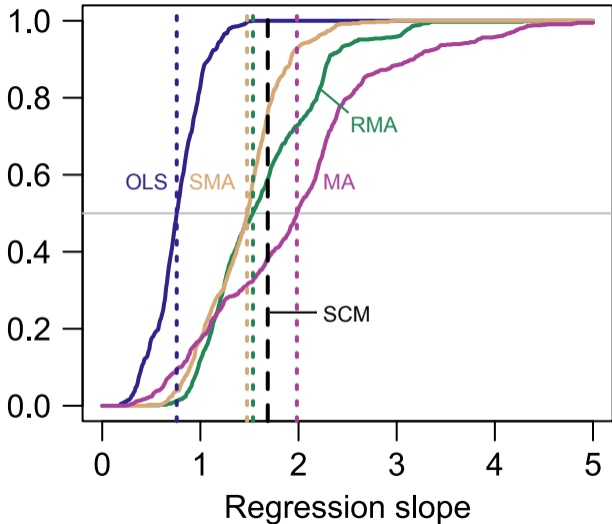

Supplement: S2 Fig — Shown are the results of major-axis (MA), scaled major-axis (SMA), and ranged major-axis (RMA) regression of protein levels on log mRNA levels, all values log-transformed but otherwise raw, with slopes extracted and shown as cumulative distributions. The SCM fit value is provided for reference (black dashed line). (PDF) [file pgen.1005206.s002.pdf]

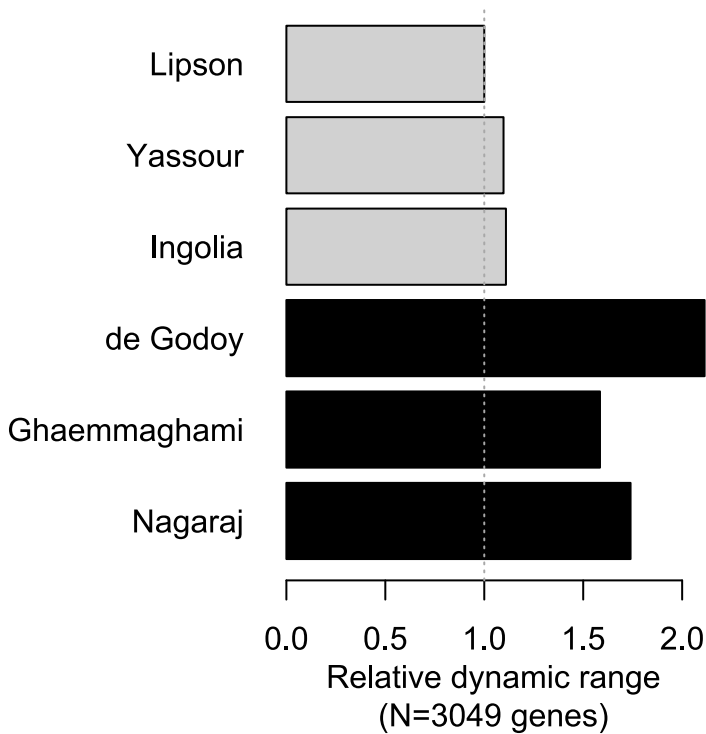

Supplement: S3 Fig — Relative dynamic ranges of large mRNA (top, gray) and protein (bottom, black) datasets. Raw data were log-transformed and the subset of genes covered by all studies were isolated (3,049 genes); the widths of the central 95% quantile of the data from each study was then calculated. To ease comparisons to results in the main text, all widths were scaled by that of the narrowest dataset. (PDF) [file pgen.1005206.s003.pdf]

Recent RNA-seq mRNA

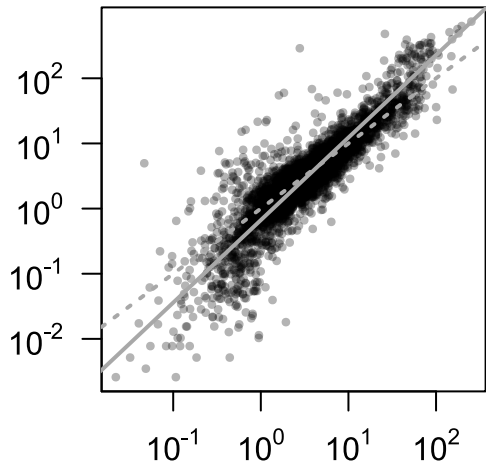

SCM mRNA

Empirical CDF

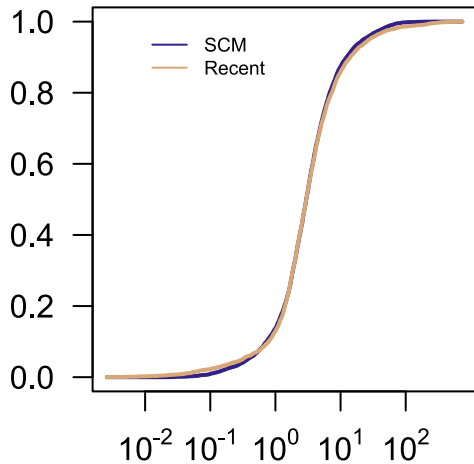

mRNA level

Supplement: S4 Fig — Left, scatterplot of recent RNA-seq data normalized to match the SCM molecules-per-cell median, against SCM estimates of mRNA levels. Dotted line shows slope of 1, solid line shows RMA slope (1.27). Right, cumulative distribution of the same data. Distributions are indistinguishable by Kolmogorov-Smirnov and Mann-Whitney U tests (P > 0.1 for both). (PDF) [file pgen.1005206.s004.pdf]
